# Causes and outcomes of non-chemotherapy induced neutropenic fever in hospitalized adults: An observational study

**DOI:** 10.1097/MD.0000000000038060

**Published:** 2024-05-03

**Authors:** Kyle G. Crooker, Eleanor R. Stedman, Juvena R. Hitt, Bradley J. Tompkins, Allen B. Repp

**Affiliations:** a Department of Medicine, University of Vermont Medical Center, Burlington, VT; b Department of Medicine, The Larner College of Medicine at the University of Vermont, Burlington, VT.

**Keywords:** adults, fever, humans, neutropenia, retrospective studies

## Abstract

Neutropenic fever in adults undergoing chemotherapy for cancer treatment is a medical emergency and has been the focus of numerous studies. However, there is a paucity of data about non-chemotherapy induced neutropenic fever (non-CINF). We retrospectively reviewed 383 adults with neutropenic fever hospitalized at one academic medical center between October 2015 and September 2020 to characterize the frequency, causes, and outcomes of non-CINF. Twenty-six percent of cases of neutropenic fever were non-chemotherapy induced. Among these, the major causes of neutropenia were hematologic malignancy, infection, and rheumatologic disease, and the major causes of fever were infections. Patients with non-CINF had a higher 30-day mortality than those with chemotherapy induced neutropenic fever (25% vs 13%, *P* = .01). Non-CINF constituted > 25% of neutropenic fever events in hospitalized adults and was associated with a high mortality rate.

## 1. Introduction

Neutropenic fever is a medical emergency and a significant cause of mortality, morbidity, and healthcare costs.^[1]^ The vast majority of information about neutropenic fever derives from studies of patients undergoing cytotoxic chemotherapy for cancer. In this context, neutropenic fever is associated with an in-hospital mortality rate of approximately 10%, excess length of stay, and increased cost of hospitalization.^[2]^ Multiple clinical practice guidelines and risk calculators address chemotherapy induced neutropenic fever (CINF).^[1,3,4]^ However, neutropenia can also be caused by non-chemotherapy drugs, infections, autoimmune conditions, and malignancies in the absence of chemotherapy.^[1]^ While much is known about CINF, there is a paucity of data on the frequency, causes, and outcomes of non-chemotherapy induced neutropenic fever (non-CINF).^[4]^ To address this knowledge gap, we sought to characterize the frequency, causes and outcomes of non-CINF in a sample of hospitalized adults.

## 2. Methods

We performed a retrospective observational study of adults hospitalized at the University of Vermont Medical Center who had neutropenic fever present at admission or who developed neutropenic fever during their hospitalization. The University of Vermont Medical Center is a 620-bed academic medical center and level 1 trauma center located in Burlington, Vermont, United States of America. We used an electronic query of health record data to identify all patients ≥ 18 years old who were discharged from October 1, 2015 to September 30, 2020 and had potential neutropenic fever. We defined potential neutropenic fever as absolute neutrophil count (ANC) < 500 cells/µL within 48 hours before or after a temperature ≥ 38.0 °C at any point during their admission. Patients under observation status, inpatient psychiatry status or acute rehabilitation status were excluded. Two of the authors performed structured manual chart review of these encounters. Study data were collected and managed using REDCap electronic data capture tools hosted at the University of Vermont.^[5]^ Chart abstraction protocols were refined until inter-rater agreement exceeded 90%. Based on chart review, the investigators determined if the case met Infectious Diseases Society of America criteria for neutropenic fever: a single temperature >38.3 °C or a temperature > 38.0 °C sustained for > 1 hour in a patient with an ANC < 500 cells/µL or an ANC expected to fall below 500 cells/µL during the subsequent 48 hours.^[3]^ A neutropenic fever event was defined as CINF if the patient had received chemotherapy or hematopoietic stem cell transplant within 42 days. A neutropenic fever event was defined as non-CINF if the patient had not received chemotherapy or hematopoietic stem cell transplant within 42 days. Chart review was conducted in a randomized fashion and continued until 100 cases of non-CINF had been identified and reviewed. The investigators categorized the causes of neutropenia and causes of fever for non-CINF events, based on the diagnoses determined by the clinical team during the hospitalization. The causes of neutropenia and fever were further classified as confirmed or suspected, based on the availability of microbiologic data or other confirmatory testing. Outcomes of in-hospital death and 30-day mortality were compared between patients with CINF and non-CINF. While the primary goal of the study was descriptive, we estimated that a total sample size of 462 patients would be needed to provide 80% power to detect a 10% difference in aggregate mortality between groups.

### 2.1. Statistical analysis

Statistical analyses were conducted in Stata 16.1 (StataCorp, LLC. 2021. College Station, TX). Data were analyzed using the Wilcoxon rank sum test for continuous variables and chi-square analysis for categorical variables.

### 2.2. Ethics statement

The Research Protections Office at the University of Vermont determined that the project was exempt from review under exemption category 4 (secondary research on data or specimens).

## 3. Results

**Figure 1.**
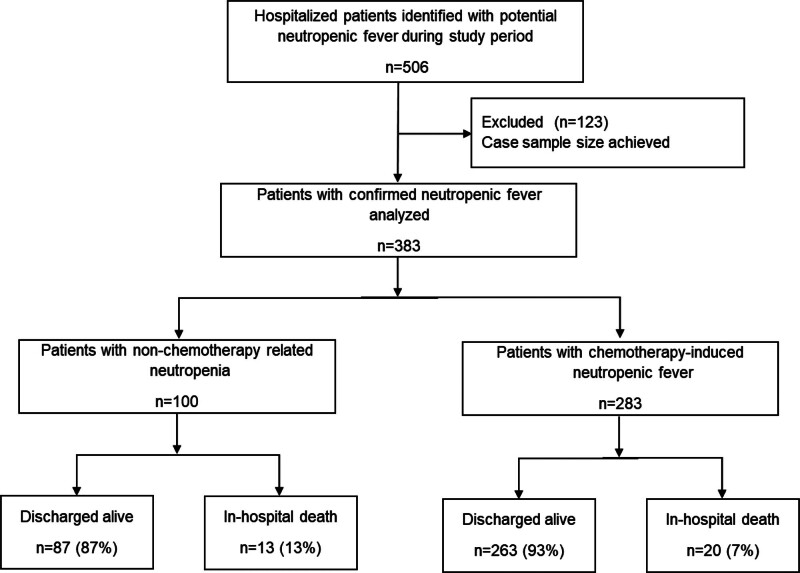
Flow diagram showing selection of records for analysis and in-hospital mortality.

A total of 506 patients were identified as potentially having neutropenic fever during the study period based on the electronic data query. Of these, 383 inpatient records were manually reviewed and confirmed to meet the Infectious Diseases Society of America definition of neutropenic fever. Among the 383 inpatients with neutropenic fever, 283 (74%) were chemotherapy induced and 100 (26%) were non-chemotherapy induced. (Fig. 1)

Among patients with non-CINF, the mean age was 61 years (Table 1). Compared with patients with CINF, the group with non-CINF had a lower percentage of female patients (39% vs 53%, *P* = .01) and a higher expected mortality (0.08 vs 0.04, *P* < .01).

**Table 1:** Characteristics of hospitalized patients with chemotherapy and non-chemotherapy induced neutropenic fever.

Characteristic	Non-chemotherapy induced neutropenic fever (n = 100)	Chemotherapy induced neutropenic fever (n = 283)	*P*-value
Age in years, mean (SD)	61 (17)	60 (16)	.61
Female, n (%)	39 (39%)	151 (53%)	.01
Vizient expected mortality, mean (SD)	0.08 (0.16)	0.04 (0.09)	<.01
Three most common MSDRGs, n (%)	1.Major hematologic/immunologic diagnosis, 36 (36%) 2.Septicemia, 18 (18%) 3.Acute leukemia, 4 (4%)	1.Major hematologic/immunologic diagnosis, 104 (37%) 2.Septicemia, 28 (10%) 3.Acute leukemia, 17 (6%)	N/A

MSDRG = medicare severity diagnosis related group, N/A = not applicable, SD = standard deviation.

### 3.1. Causes of neutropenia among patients with non-CINF

Out of the 100 patients with non-CINF, 93 had a confirmed or suspected etiology of neutropenia, with some having multiple contributing causes, resulting in a total of 111 identified causes of neutropenia (Table 2). Hematologic malignancy (43/100, 43%), non-chemotherapy medications (24/100, 24%), infection (16/100, 16%), and rheumatologic disease (15/100, 15%) were the most common etiologies of neutropenia in this group of patients. The supplemental table details the specific identified causes of neutropenia.

**Table 2:** Causes of neutropenia and fever in hospitalized adults with non-chemotherapy induced neutropenic fever.

	Causes of neutropenia			Causes of fever		
Cause	Confirmed	Suspected	Total	Confirmed	Suspected	Total
Infection, total	3	13	16	46	44	90
Bacterial	0	9	9	29	44	73
Viral	3	4	7	9	0	9
Fungal	0	0	0	6	0	6
Mycobacterial	0	0	0	2	0	2
Malignancy, solid	0	0	0	0	1	1
Malignancy, hematologic	32	11	43	1	0	1
Alcohol/Illicit substances	0	1	1	0	1	1
Non-chemotherapy medications	9	15	24	0	2	2
Rheumatologic	12	3	15	0	4	4
Thrombosis	0	0	0	0	1	1
Other	5	7	12	0	2	2
Total	61	50	111	47	54	101

Hematologic malignancies were the most frequent cause of neutropenia in patients with non-CINF. The most common hematologic malignancies associated with neutropenia were myelodysplastic syndrome (19/100, 19%) followed by acute myeloid leukemia (13/100, 13%). Chronic lymphocytic leukemia (2/100, 2%) and large granular lymphocytic leukemia (2/100, 2%) were the next most common malignancies identified as causes of neutropenia (Table S1, Supplemental Digital Content, http://links.lww.com/MD/M406). No cases of neutropenia were attributed to solid malignancies.

Non-chemotherapy medications were cited as a cause of neutropenia in 24/100 patients (24%) with non-CINF. The non-chemotherapy medication classes determined to cause neutropenia included immunosuppressives (12/100, 12%), antimicrobials (7/100, 7%), and anticonvulsants (2/100, 2%). A wide range of antimicrobials accounted for cases of neutropenia including the antiviral medication valganciclovir (2/100, 2%), and common antibiotics such as cephalosporins (cefazolin 1/100, 1%; ceftazidime 1/100, 1%), levofloxacin (1/100, 1%), piperacillin-tazobactam (1/100, 1%), and trimethoprim-sulfamethoxazole (1/100, 1%).

Infection was identified as a cause of neutropenia in 14 non-CINF cases (14%). The infectious causes were divided among bacterial (8/100, 8%) and viral (8/100, 8%). Human immunodeficiency virus was implicated as the cause of neutropenia in 4 cases (4%). The most frequent bacterial causes of neutropenia were *E. coli* (2/100, 2%) and *P. aeruginosa* (2/100, 2%). No fungal or mycobacterial etiologies were identified.

The most common rheumatologic causes of neutropenia in patients with non-CINF were autoimmune neutropenia (8/100, 8%) and common variable immunodeficiency (3/100, 3%). Felty syndrome and systemic lupus erythematosus each contributed to 2 cases (2/100, 2% for each).

### 3.2. Causes of fever among patients with non-CINF

Among the 100 patients with non-CINF, 87 had a suspected or confirmed etiology of fever, with some having multiple contributing causes, resulting in a total of 101 identified causes of fever (Table 2). Infection was the most common etiology of fever (90/100, 90%), including bacterial (73/100, 73%), viral (9/100, 9%), and fungal (6/100, 6%) organisms. Within infectious causes of fever, *Staphylococcus aureus* (6/100, 6%), Streptococcus species (5/100, 5%), *Pseudomonas aeruginosa* (4/100, 4%), *Escherichia coli* (3/100, 3%) and cytomegalovirus (3/100, 3%) were the specific organisms most frequently identified.

Rheumatologic conditions were determined to cause fever in 4 cases (4%), including systemic lupus erythematosus in 2 cases (2%) and lupus-like syndrome in 1 case (1%). Non-chemotherapy medications were established as the etiology of fever in 2 cases (2%), both attributed to anti-thymocyte globulin. Other etiologies of fever in this sample of patients included deep venous thrombosis (1/100, 1%), hemophagocytic lymphohistiocytosis (1/100, 1%), and status epilepticus (1/100, 1%). The supplemental table details the specific identified causes of fever.

### 3.3. Outcomes of non-CINF

Of the 100 non-CINF patients, 24 expired in the hospital or were discharged to hospice. Compared to patients with CINF, patients with non-CINF had a similar median length of stay (7.5 days vs 9 days, *P* = .96) but a higher aggregate 30-day mortality (25% vs 13%, *P* = .01) (Table 3).

**Table 3:** Outcomes of hospitalized patients with chemotherapy and non-chemotherapy induced neutropenic fever

Outcome	Chemotherapy	Non-chemotherapy	*P*-value
Length of stay, median (IQR)	9 (5,18)	7.5 (4,12)	.96
In-hospital mortality, n (%)	20 (7%)	13 (13%)	.07
Post-discharge 30-day mortality, n (%)	19 (7%)	12 (12%)	.10
Aggregate 30-day mortality, n (%)	39 (13%)	25 (25%)	.01

IQR = interquartile range.

## 4. Discussion

Non-CINF has not been well-studied or characterized previously. In this observational study, non-CINF constituted more than 25% of neutropenic fever events in hospitalized adults and was associated with a markedly high mortality. The most identified causes of neutropenia were hematologic malignancy, infection, rheumatologic disease, and medications. Bacterial infections were the predominant cause of fever.

In our study, hematologic malignancy was the most common cause of neutropenia among patients with non-CINF. Other studies investigating the causes of new-onset pancytopenia in adults have also identified myelodysplastic syndrome, acute myeloid leukemia and chronic lymphocytic leukemia as culprits.^[6,7]^ Although hematologic malignancy is a known cause of neutropenia in the absence of chemotherapy, prior studies of fever in neutropenic patients with hematologic malignancies have focused on patients treated with chemotherapy, while the present study focused on patients who had not received chemotherapy.^[1,8]^

In our sample, infections accounted for neutropenia in 14% of the non-CINF cases. In comparison, a 2006 study reported infectious etiologies in 9% of adults with neutropenia incidentally discovered on laboratory testing.^[9]^ We found half of the infectious causes of neutropenia were viruses and the other half were bacteria. Human immunodeficiency virus, a well-established cause of neutropenia, was the most common viral culprit in our study.^[10,11]^ Other literature has also described the relationship between bacterial infections and neutropenia, especially in the setting of sepsis.^[12]^ A broad array of bacterial infections have been associated with mild to moderate neutropenia, with tuberculosis, typhoid fever, tularemia, shigellosis, brucellosis, and rickettsial infections as classic examples.^[12,13]^ These infections less frequently induce severe neutropenia, and these organisms were not identified as causes of neutropenia in our sample of patients with severe neutropenia (ANC < 500 cells/µL). Our finding of infections as the cause of fever in 89% of non-CINF patients is higher than the 74% estimated within generalized hospital patients.^[14]^ It is not a surprising finding, since the patients in our study had neutropenia, which was caused by infection in some patients and likely increased susceptibility to infection in other patients.^[15]^

Rheumatologic disease was shown to cause both neutropenia and fever. Neutropenia is commonly seen in systemic autoimmune conditions including autoimmune neutropenia, systemic lupus erythematosus, and Felty syndrome associated with rheumatoid arthritis.^[1,13,15]^ Moreover, fever is common in rheumatologic diseases: 50% of patients with active systemic lupus erythematosus experience fever.^[16]^

Immunosuppressives were the most commonly identified medication class to cause neutropenia in our study. Even though these medications were not used as cancer chemotherapy, it might be expected that they were a common culprit for neutropenia as this adverse effect has been well established.^[17]^ The antimicrobial medication class has also been implicated as a frequent cause of neutropenia.^[18]^ In our study, a wide range of antibiotics were documented as causes of neutropenia, among which beta-lactams comprised approximately half. These findings are consistent with prior studies showing that beta-lactams are responsible for nearly 50% of antimicrobial induced neutropenia.^[18]^ The extensive list of non-chemotherapy medications causing neutropenia identified in this study are compatible with findings of other observational studies.^[4,18,19]^ Surprisingly, the only medication pinpointed as a cause of fever in our study was anti-thymocyte globulin, but prior studies describe fever related to drugs as relatively common.^[20]^ The only illicit drug implicated in causing neutropenia in our study was heroin. Although heroin is not typically associated with neutropenia, adulterants in the illicit drug supply like levamisole are a growing cause of neutropenia and may have contributed to neutropenia in the single case in our sample.^[21]^

There are guidelines for evaluation and management of neutropenic fever, but these guidelines are based exclusively on studies in patients with CINF and do not address patients with non-CINF.^[3]^ A significant percentage of hospitalized adults with neutropenic fever in this study had non-CINF, and this patient population may benefit from alternative approaches to evaluation and management. The high mortality observed in patients with non-CINF highlights the need for clinicians to recognize the severity of this condition. The 25% aggregate 30-day mortality in our sample of patients with non-CINF was considerably higher than the 13% 30-day mortality in patients who had received chemotherapy. Of note, the 7% in-hospital mortality observed in CINF patients included in this study is comparable to estimates of in-hospital CINF mortality reported in the literature.^[2]^

There are important limitations to this study. It was completed at one academic medical center, which may limit its generalizability. Inherent to observational studies, there may be unmeasured confounders that account for the differences in observed outcomes between hospitalized patients with non-CINF vs CINF. Moreover, reliance primarily on physician documentation may have led to the inclusion of inaccurate or incomplete etiologies of fever and neutropenia.

## 5. Conclusion

This study offers novel insights into non-CINF. Our findings show that patients with non-CINF are distinct from the population of patients with CINF, with different characteristics, risk profiles, and outcomes. This investigation only begins to fill the void within the literature about non-CINF. However, it importantly identifies that non-CINF makes up a sizeable portion of neutropenic fever events in hospitalized adults and is associated with high mortality. In light of these findings, greater efforts are needed to study non-CINF and develop clinical practices to prevent and manage this condition.

## Author contributions

**Conceptualization:** Kyle G. Crooker, Allen B. Repp.

**Data curation:** Kyle G. Crooker, Juvena R. Hitt, Bradley J. Tompkins, Allen B. Repp.

**Formal analysis:** Kyle G. Crooker, Juvena R. Hitt, Bradley J. Tompkins, Allen B. Repp.

**Investigation:** Kyle G. Crooker, Eleanor R. Stedman, Allen B. Repp.

**Methodology:** Kyle G. Crooker, Eleanor R. Stedman, Allen B. Repp.

**Project administration:** Kyle G. Crooker, Eleanor R. Stedman, Juvena R. Hitt, Bradley J. Tompkins, Allen B. Repp.

**Resources:** Juvena R. Hitt, Bradley J. Tompkins.

**Software:** Bradley J. Tompkins.

**Supervision:** Allen B. Repp.

**Writing – original draft:** Kyle G. Crooker.

**Writing – review & editing:** Kyle G. Crooker, Eleanor R. Stedman, Juvena R. Hitt, Bradley J. Tompkins, Allen B. Repp.

## Supplementary Material


