# Supplementary material for: Causes and outcomes of non-chemotherapy induced neutropenic fever in hospitalized adults: An observational study
Source: Medicine (Baltimore). 2024 May 3;103(18):e38060. doi: 10.1097/MD.0000000000038060 (PMC11062702; doi:10.1097/MD.0000000000038060)
Supplement: Supplementary file 1 [file medi-103-e38060-s001.docx]

**Supplemental Table** Specific Causes of Neutropenia and Fever in Hospitalized Adults with Non-chemotherapy Induced Neutropenic Fever

|  | Cause of Neutropenia (count) | Cause of Fever (count) |
| --- | --- | --- |
| Bacterial | *E. coli* (2)  *P. aeruginosa* (2)  Suspected bacteria unidentified (2)  *E. meningoseptica* (1)  *S. pneumonia* (1) | Methicillin susceptible *S. aureus* (6)  Streptococcus species (5)  *P. aeruginosa* (4)  *E. coli* (3)  Methicillin resistant *S. aureus* (2)  *A. baumanni* (1)  *C. difficile* (1)  *C. septicum* (1)  Coagulase negative staphylococcus (1)  *E. cloacae* (1)  *E. faecalis* (1)  *E. meningoseptica*  Lactobacillus species (1)  Leptotrichia species (1)  Neurosyphilis (1)  Serratia species (1) |
| Viral | Human immunodeficiency virus (4)  Cytomegalovirus (1)  Influenza A (1)  Rhinovirus (1)  Suspected virus unidentified (1) | Cytomegalovirus (3)  Adenovirus (1)  Hepatitis B virus (1)  Metapneumovirus (1)  Rhinovirus (1)  SARS-CoV-2 (1) |
| Fungal | None | Candida species (3)  *P. jirovecii* (2)  Aspergillus species (1) |
| Mycobacterial | None | *Mycobacterium avium* complex (1) |
| Malignancy, solid | None | Unspecified lung malignancy (1) |
| Malignancy, hematologic | Myelodysplastic syndrome (19)  Acute myeloid leukemia (13)  Chronic lymphocytic leukemia (2)  Large granular lymphocyte leukemia (2)  Acute lymphoblastic leukemia (1)  Chronic eosinophilic leukemia (1)  Diffuse large b-cell lymphoma (1)  Mantle cell lymphoma (1)  Myelofibrosis (1)  Small lymphocytic lymphoma (1) | Central nervous system lymphoma (1) |
| Alcohol/Illicit substances | Heroin (1) | None |
| Non-chemotherapy medications classes | Immunosuppressant (12)  Antimicrobial (7)  Anticonvulsant (2)  Antiarrhythmic (1)  Antihypertensive (1)  Antithyroid (1) | Immunosuppressant (2) |
| Non-chemotherapy medications | Methotrexate (3)  6-mercaptopurine (2)  Mycophenolate mofetil (2)  Valganciclovir (2)  Azathioprine (1)  Cefazolin (1)  Ceftazidime (1)  Clozapine (1)  Digoxin (1)  Hydrochlorothiazide (1)  Infliximab (1)  Levofloxacin (1)  Methimazole (1)  Ocrelizumab (1)  Piperacillin-tazobactam (1)  Rituximab (1)  Tacrolimus (1)  Trimethoprim-sulfamethoxazole (1)  Valproic acid (1) | Antithymocyte globulin (2) |
| Rheumatologic | Autoimmune neutropenia (8)  Common variable immunodeficiency (3)  Felty syndrome (2)  Systemic lupus erythematosus (2) | Systemic lupus erythematosus (2)  Cryptogenic organizing pneumonia (1)  Lupus-like syndrome (1) |
| Thrombosis | None | Deep venous thrombosis (1) |
| Other | Aplastic anemia (6)  Acute respiratory distress syndrome (1)  Cirrhosis (1)  Evans syndrome (1)  Fucoidan supplement (1)  Marrow hypoperfusion (1)  Vitamin B12 deficiency (1) | Hemophagocytic lymphohistiocytosis (1)  Status epilepticus (1) |
